# The Reconstruction of Post-traumatic Cranial Bone Depression by Duplicating an Autogenous Bone Flap With a Prosthetic Polymethyl Methacrylate Cranial Stent

**DOI:** 10.7759/cureus.65353

**Published:** 2024-07-25

**Authors:** Ankita Pathak, Mithilesh M Dhamande, Sweta G Pisulkar, Surekha A Dubey, Anjali Bhoyar, Arushi Beri, Prasanna R Sonar

**Affiliations:** 1 Department of Prosthodontics, Sharad Pawar Dental College and Hospital, Datta Meghe Institute of Higher Education and Research, Wardha, IND; 2 Department of Oral Medicine and Radiology, Sharad Pawar Dental College and Hospital, Datta Meghe Institute of Higher Education and Research, Wardha, IND

**Keywords:** maxillofacial prosthetics, neurosurgery, pmma, cranioplasty, cranial stent

## Abstract

Cranioplasty is a common surgical procedure used to restore the shape of the calvaria. Autogenous bone flaps provide biological repair with the least morbidity to the donor location. One method for reusing bone autograft during cranioplasty involves low-temperature preservation followed by autoclaving. According to the literature, there was a good to modest improvement in the skull's symmetry, cosmesis, scars, and contour. It was determined that autoclaved frozen autogenous cerebral bone flaps are a safe and reliable method of cranioplasty. Despite its apparent simplicity, cranioplasty has been found to carry a relatively high complication rate, ranging from 12% to 50%, which starkly contrasts with the standard elective craniotomy, with a complication rate typically ranging from 24% to 60%. Complications associated with autogenous bone flap procedures are varied. The presented case report primarily focuses on the duplication of autogenous bone flaps by using clear polymethyl methacrylate.

## Introduction

Cranioplasty is used to restore the form and function of the calvaria. It is typically carried out after a decompressive craniectomy (DC) for a brain injury or after surgery to remove a tumor [1]. In general, abnormalities of the cranium are categorized as congenital or acquired. Since skull reconstruction is in high demand, the frequency of cranial accidents has significantly grown. Rehabilitation for these abnormalities has, therefore, increased as well. In addition to addressing psychological concerns and restoring appearance, surgical correction or repair, or cranioplasty, also attempts to improve social acceptance and overall performance [2,3]. Elliott and Scott [4] first reported on using frozen autogenous bone for cranial reconstruction in 1951. Before reconstruction, the frozen autogenous bone graft can be autoclaved, radiotreated, or submerged in a sterile solution as a cranioplasty material [5,6]. Subcutaneous preservation of craniectomy bone flaps typically occurs in the patient's lateral portion of the thigh and anterior abdominal wall [6,7]. Sometimes, however, subcutaneous preservation of autologous bone is warranted due to the patient's poor systemic health and related comorbidities. Other approaches for storing autogenous bone grafts include freezing, cryogenic preservation, and preservation in bactericidal solutions [8-11]. Cranioplasty was completed by autoclaving the bone for 40 minutes at 121°C under 15 psi after it had been thawed at room temperature. Autogenous bone graft storage choices are typically restricted in hospitals without sufficient bone bank facilities [1].

In addition to being a protective and esthetic procedure, replacing the skull has curative advantages. Although the likelihood of surgical site infections is low with this approach, the rate of bone flap resorption is high. Seizures, extradural hemorrhage, subdural hygroma, wound infection, bone flap resorption, wound dehiscence, and removal of the bone flap due to brain edema were among the complications [12]. Bone flap resorption was the most common complication [12]. Because the esthetic results are good, neurosurgeons often use autologous bone flaps for cranial reconstruction. Hence, to avoid the above-mentioned complications and achieve excellent aesthetic results, cranial reconstruction was done by duplicating the autogenous bone flap with polymethyl methacrylate (PMMA) in the presented case report.

## Case presentation

**Figure 1:**
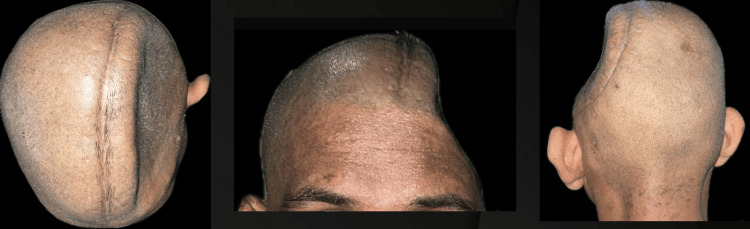
Preoperative evaluation of the cranial defect

A 30-year-old male patient reported to the Department of Neurosurgery with the primary complaint of calvarial deformity (Figure 1). Upon complete examination and history taking, it was observed that the patient had met with a motor vehicle accident (MVA).

**Figure 2:**
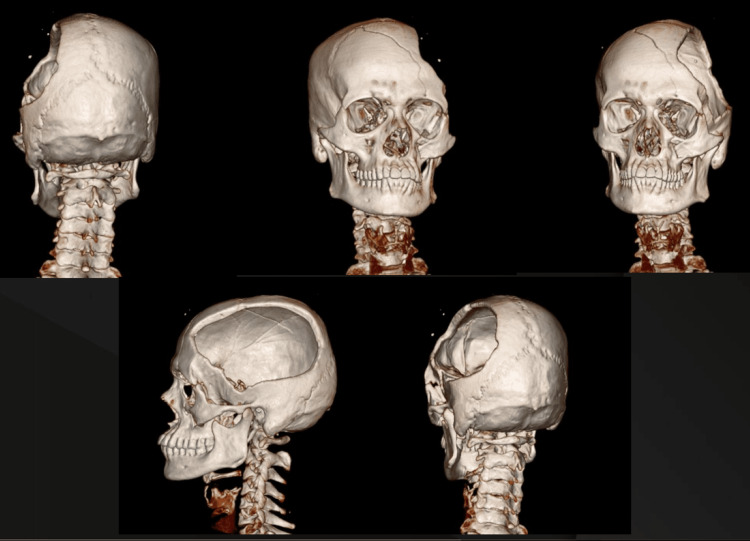
Reconstructed 3D images from CT scan

In the history of the present illness, it was noted that the patient was asymptomatic six months prior. Then, he met with the MVA, which resulted in several bone fractures, including the cranium. CT scan was performed to evaluate the defect site (Figure 2).

**Figure 3:**
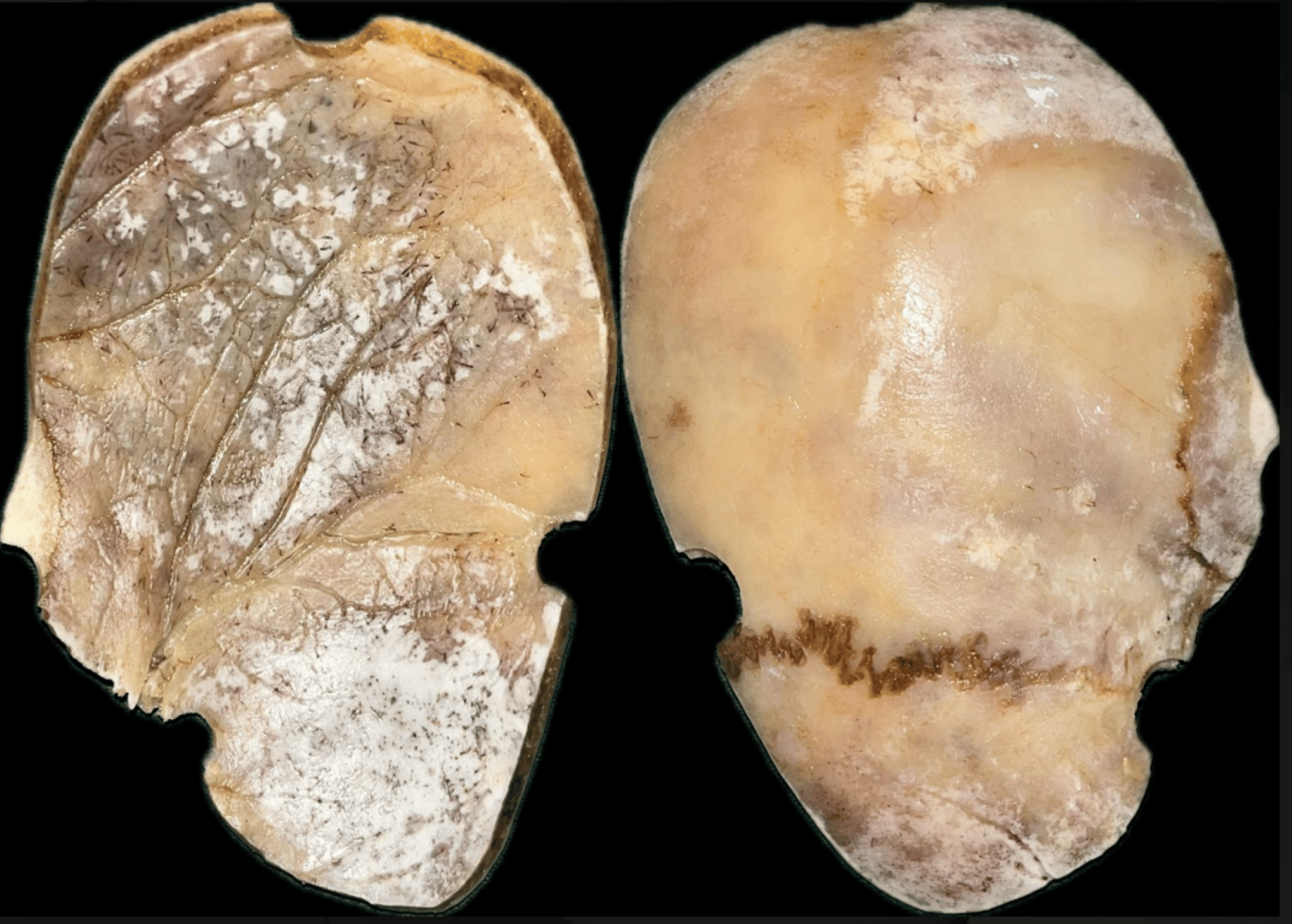
Removed left-side bone flap of the patient

The bone flap, consisting of the left frontal, parietal, and temporal bones, was removed due to fracture and secondary infection (Figure 3). It was preserved in a deep freezer.

Utilizing the autogenous bone flap was avoided as it had been stored longer in an unfavorable environment, which may have led to postsurgical complications. Upon evaluation, the defect was 13 cm × 6 cm × 6 cm. Before duplicating the bone flap, the flap was adapted to the defect site to assess its fit on the defect. To replicate the exact anatomy of the skull, it was decided to duplicate the autogenous bone with alloplastic material.

**Figure 4:**
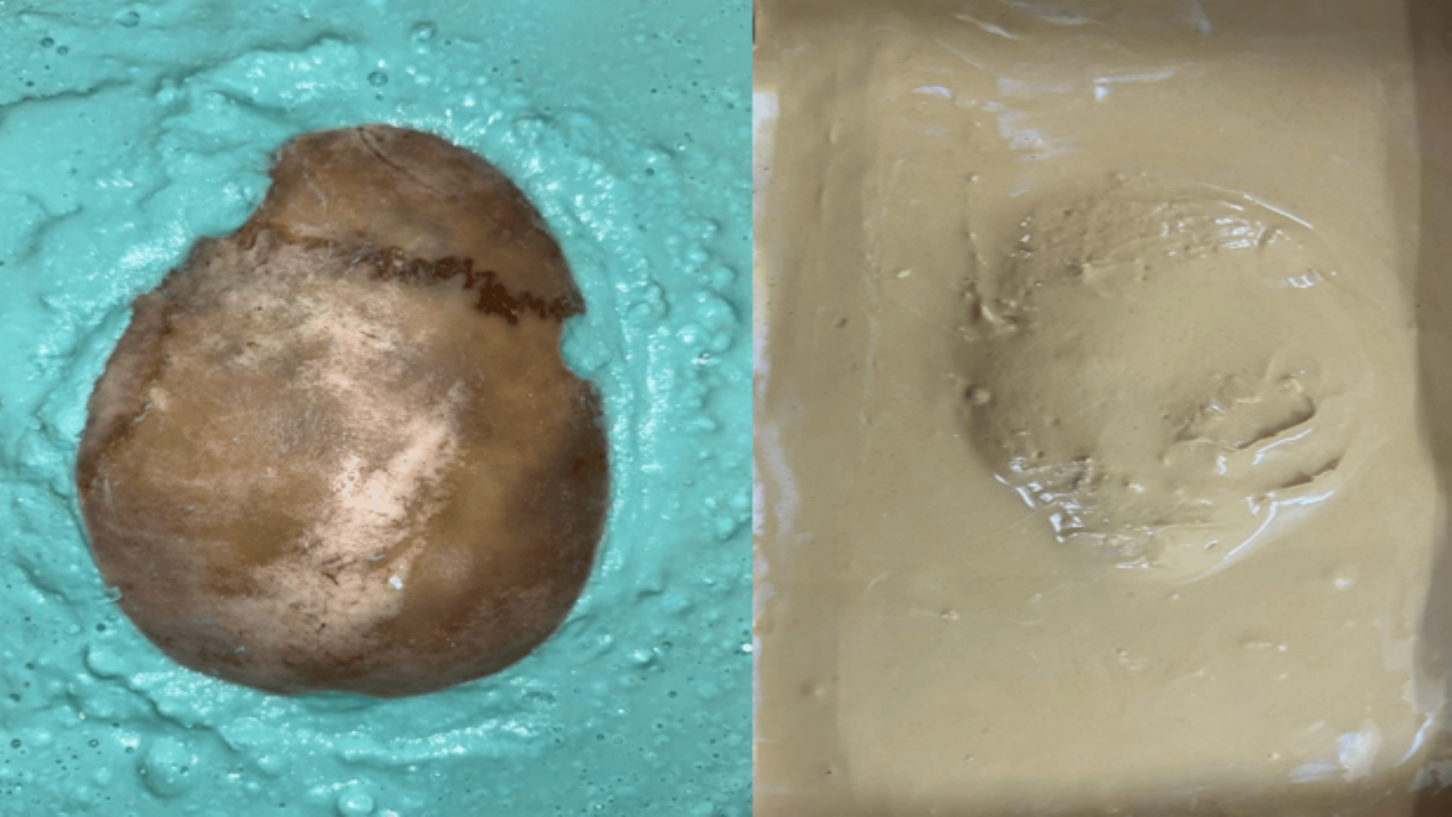
Investing a bone flap in the mold

**Figure 5:**
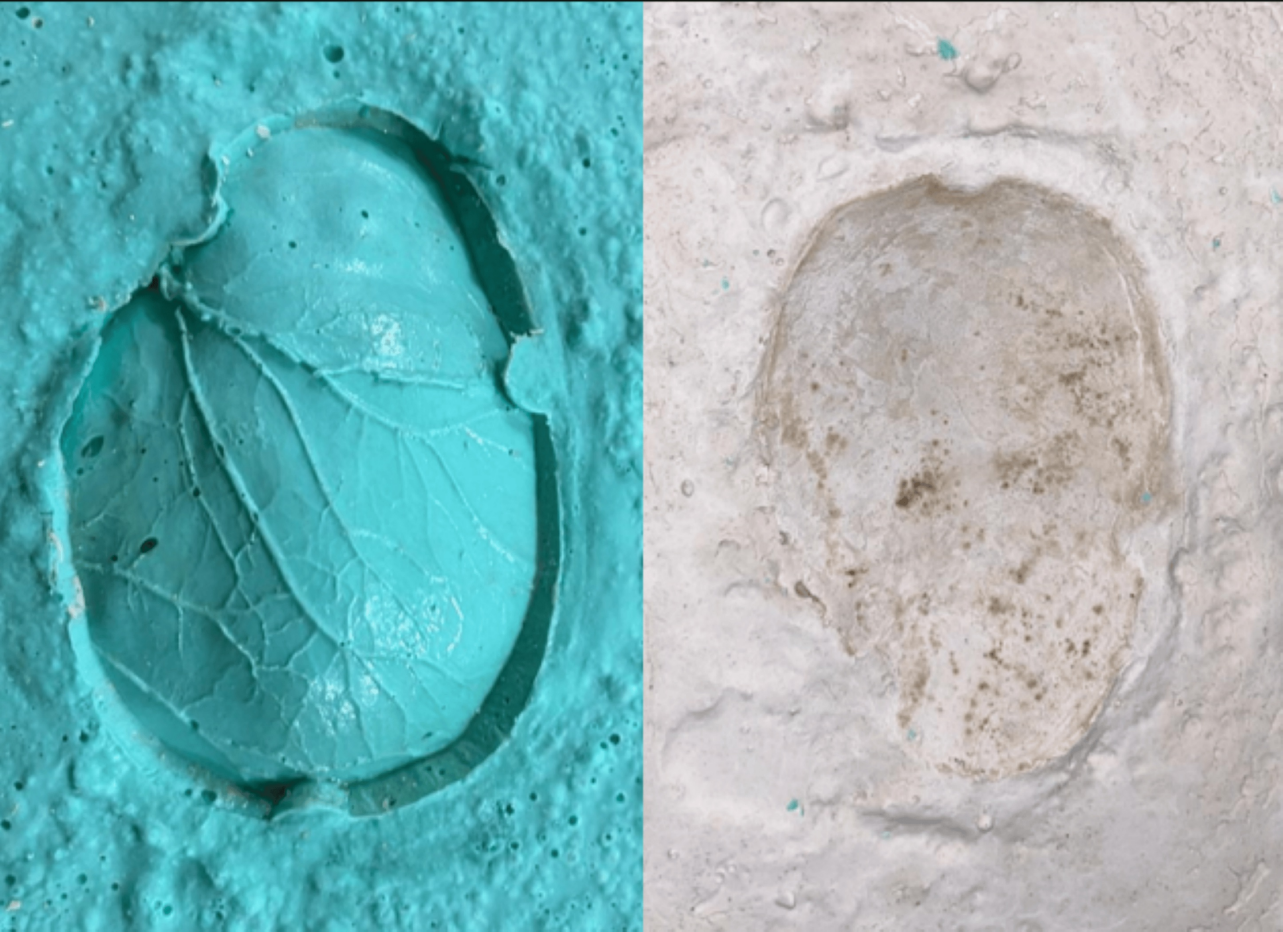
Mold cavity for the fabrication of the wax pattern

**Figure 6:**
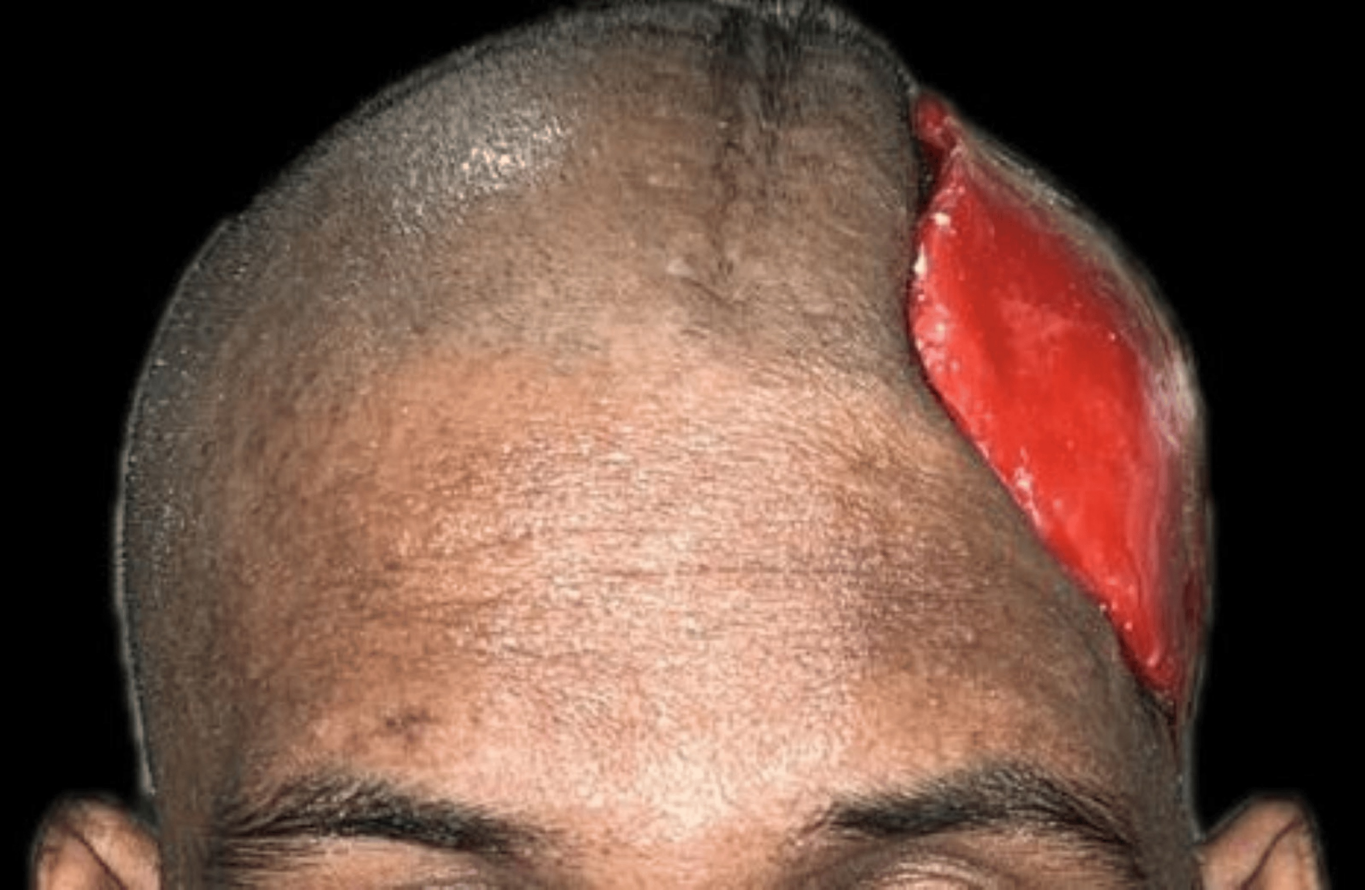
Wax trial

**Figure 7:**
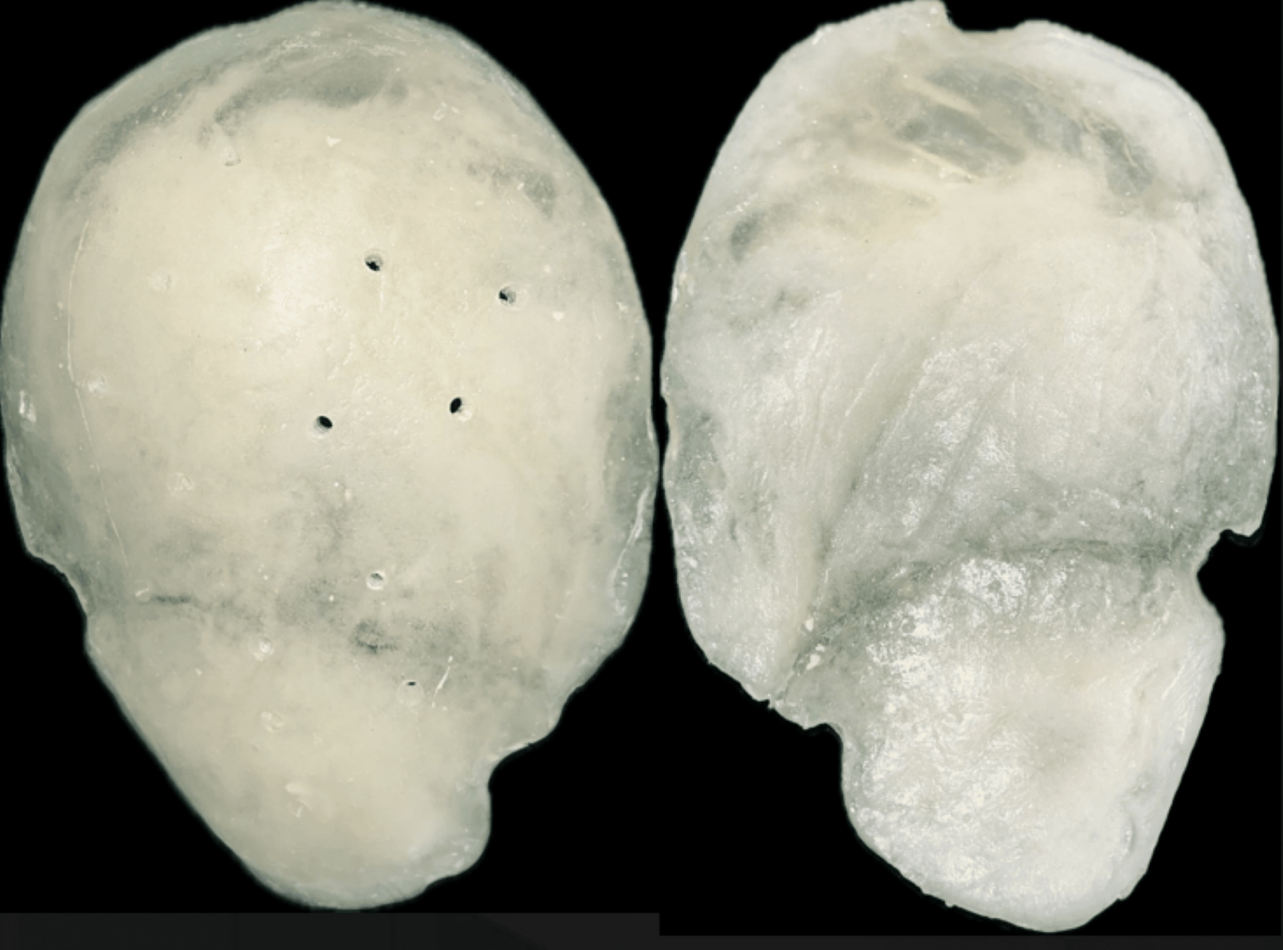
Fabricated cranial stent

The prosthetic and surgical phases encompassed the process of cranial prosthesis fabrication. In the prosthetic phase, the flap was invested in irreversible hydrocolloid material (Figure 4). After the initial set, two sections were separated, and the bone flap was removed. Modeling wax was heated and flowed in the prepared mold to fabricate the wax pattern (Figure 5). A wax trial was conducted on the patient’s defect site (Figure 6). Freehand sculpting was done in the required regions. A mold with dental plaster was fabricated using the wax pattern to ensure an accurate fit of the wax pattern. The wax pattern was removed following the initial set of plaster. The cranial stent was packed with PMMA heat-cured clear acrylic material (Figure 7). Finishing and polishing were done after retrieval of the cranial stent from the plastered mold. To reduce intracranial pressure, 8-10 vent holes were meticulously drilled in the cranial stent.

An overall thickness of 3 mm was preserved, and the PMMA cranial prosthesis's edges were beveled to 45°, by which a snug fit with the bone was obtained. Following verification, it was used intraoperatively, cleaned, and kept for around 24 hours in a 2% glutaraldehyde solution. The prosthesis's perforations facilitated the removal of inflammatory exudates and supplied blood to the flap tissues that overlay them, primarily the scalp. The prosthesis's smooth dorsal surface made it presentable and enabled a correct fit.

**Figure 8:**
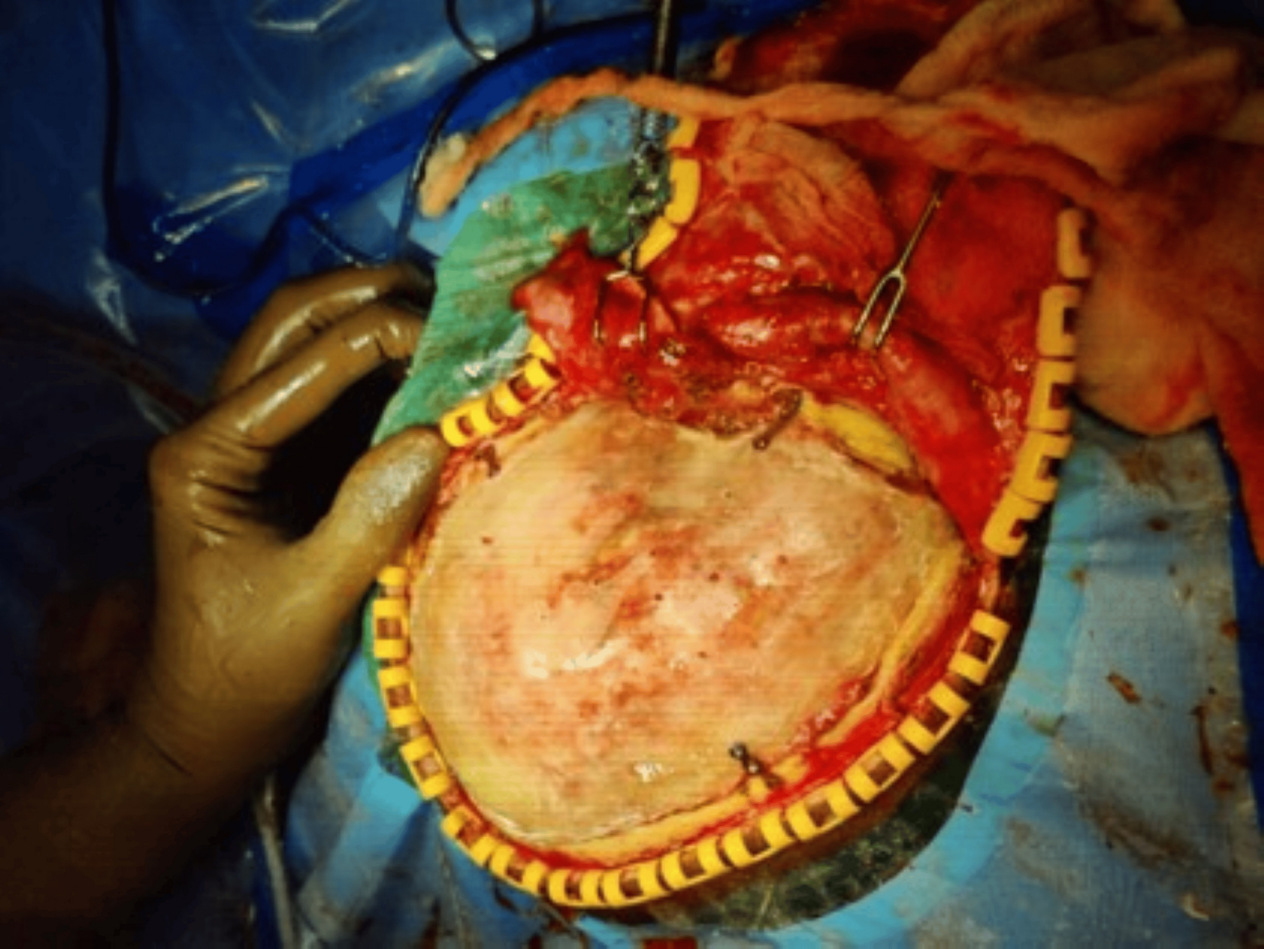
Cranioplasty with the PMMA cranial stent PMMA: polymethyl methacrylate

The cranioplasty was conducted in compliance with the guidelines set by the institution (Figure 8). Along the prior incision, the skin was opened, and then, the bony borders, (neo-)dura, and temporalis muscle flap were prepared. Four four-hole plates and self-tapping titanium screws were used to secure the miniplates on the cranial stent. Miniplates were used because of the properties of biocompatibility and osseointegration. Miniplates have demonstrated superiority over wires, requiring a 40% reduction in time during operations. Many resorbable sutures for retention were used to connect the dura to the stent to prevent the accumulation of epidural fluid. The wound was closed after inserting one or two subgaleal drain drains with suction.

**Figure 9:**
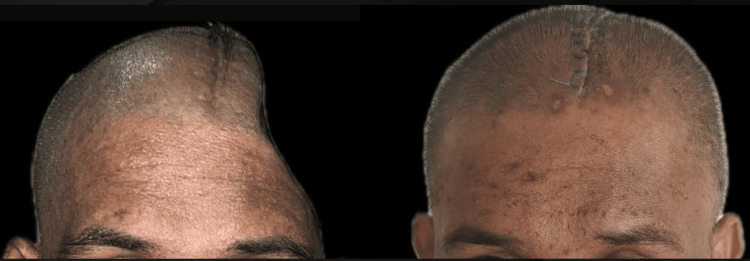
Preoperative and postoperative results

One week after surgery, the patient was evaluated in the prosthodontics department, and the patient's skull contour had significantly improved, as shown in Figure 9. The patient expressed great satisfaction with the aesthetics he received and the relief from his terrible headache and psychological anguish. After four months, the patient was evaluated and went about his social life as usual.

## Discussion

For contaminated bone flaps, replacement following disinfection is a suitable alternative that avoids the expense and time of cranioplasty [13]. Bone storage necessitates freezing temperatures or subcutaneous preservation. Neurosurgeons commonly use autologous bone flaps for cranial reconstruction due to the good esthetic outcomes that come at no extra cost. However, postoperative infection is a significant risk associated with cranioplasty utilizing frozen bone flaps. Acute bone flap infection processes start with contamination from extrinsic variables, extended surgical manipulation, wound rupture, or intrinsic factors like the autologous bone flap. It is not appropriate to classify cranioplasty utilizing autologous bone flaps as "clean" surgery, and surgical drainage in possibly contaminated operating areas needs to be assessed [14].

Facial disfigurement significantly impacts an individual's mental state in addition to its effects on appearance and functionality. An asymmetrical look results from the absence of any facial organs, which can cause social dilemmas for the patient [15-18].

Long-term research indicates that for patients who have had DC, autologous cranioplasty has been the gold standard for decades. One of the most popular storage methods is the cryopreservation of the cranial bone flap at very low temperatures (-70°C to -80°C). The cranial bone flap was kept in a refrigerator's freezer at -18°C [19]. A study was conducted to evaluate the risk factors of cryopreservation of autogenous bone flap in povidone-iodine. Short-term postoperative problems were noted to include infection in the flap, subcutaneous or extradural seroma collection, and epidural hematoma. Two patients who passed away within two years and eight patients whose flaps developed an infection and required removal were not included in the follow-up analysis. CT demonstrated bone resorption [11].

However, in the presented case report, the patient could not mention any history about the duration or how the bone flap was preserved. To avoid any complications, a duplicated, prosthetically fabricated PMMA cranial stent was decided to be used.

As the frequency of DCs rises, so does the number of cranioplasties performed to replace the resulting bone deficiency. An autologous bone flap that had been sterilized was used in a study to assess the morbidity related to cranioplasty [12].

Materials have been employed to treat cranial abnormalities, including polyetheretherketone, titanium, hydroxyapatite, methyl methacrylate, and alumina ceramics [20]. Although acrylic resins were the preferred material for cranioplasty during World War II, their usefulness in dental prostheses was starting to gain their respect. Following its discovery in 1939, methyl methacrylate underwent a great deal of experimentation in the 1940s [21]. After further investigation, it was discovered that acrylic is stronger, more heat resistant, radiolucent, and more inert than metal [22].

The residual monomer from cold polymerization may be hazardous, and PMMA has a high risk of extrusion, breakdown, and infection despite its benefits [23]. When PMMA is prepared for usage, an exothermic reaction is produced when it reacts with a monomer. This turns the material into a pliable paste and increases the risk of burns. Long-term protection is limited by the possible fragmentation of PMMA, which may cause infection and inflammatory responses. In their investigation of the long-term effects of PMMA use for cranioplasty, Blum et al. discovered a 23% complication rate within eight years following surgery. Infections made up the majority of the complications [23]. These results were further supported by Shah et al., who also showed that PMMA has a high infection rate of 12.7% [20]. Because of its exceptional tensile strength, methyl methacrylate is a synthetic material that is frequently utilized; however, over time, its susceptibility to fracture and infection rates has rendered it a less feasible material [20,24].

## Conclusions

The presented case study demonstrates the repair of a cranial defect by duplicating the autogenous bone flap. A common surgery used to restore the form and shape of the calvaria is cranioplasty. Prosthetic and surgical phases encompassed the process of making the cranial prosthesis fabrication. Further, this study also highlights the properties of PMMA and its biocompatibility with the calvarial site. The most popular material for cranial reconstruction in cases with cranial malformations is PMMA.

## References

[1] Sahoo NK, Thakral A, Janjani L (2019). Cranioplasty with autogenous frozen and autoclaved bone: management and treatment outcomes. J Craniofac Surg.

[2] Beri A, Pisulkar SG, Iratwar S, Bansod A, Jain R, Shrivastava A (2024). Revolutionizing neurosurgery: the cutting-edge era of digitally fabricated cranial stents. Cureus.

[3] Beri A, Pisulkar SK, Bagde AD, Bansod A, Dahihandekar C, Paikrao B (2022). Evaluation of accuracy of photogrammetry with 3D scanning and conventional impression method for craniomaxillofacial defects using a software analysis. Trials.

[4] Elliott H, Scott HJ (1951). The bone bank in neurosurgery. Br J Surg.

[5] Odom GL, Woodhall B, Wrenn FR (1952). The use of refrigerated autogenous bone flaps for cranioplasty. J Neurosurg.

[6] Crotti FM, Mangiagalli EP (1979). Cranial defects repair by replacing bone flaps. J Neurosurg Sci.

[7] Nakajima T, Tanaka M, Someda K, Matsumura H (1975). Subcutaneous preservation of free skull bone flap taken out in decompressive craniectomy (author's transl). [Article in Japanese]. No Shinkei Geka.

[8] Movassaghi K, Ver Halen J, Ganchi P, Amin-Hanjani S, Mesa J, Yaremchuk MJ (2006). Cranioplasty with subcutaneously preserved autologous bone grafts. Plast Reconstr Surg.

[9] Kurokawa Y, Watanabe K, Abiko S, Okamura T (1995). Cranioplasty results using decompressed bone flaps preserved in 80% ethanol. [Article in Japanese]. Jpn J Neurosurg.

[10] Fan MC, Wang QL, Sun P, Zhan SH, Guo P, Deng WS, Dong Q (2018). Cryopreservation of autologous cranial bone flaps for cranioplasty: a large sample retrospective study. World Neurosurg.

[11] Zhang J, Peng F, Liu Z, Luan J, Liu X, Fei C, Heng X (2017). Cranioplasty with autogenous bone flaps cryopreserved in povidone iodine: a long-term follow-up study. J Neurosurg.

[12] Mracek J, Hommerova J, Mork J, Richtr P, Priban V (2015). Complications of cranioplasty using a bone flap sterilised by autoclaving following decompressive craniectomy. Acta Neurochir (Wien).

[13] Jankowitz BT, Kondziolka DS (2006). When the bone flap hits the floor. Neurosurgery.

[14] Huang YH, Yang TM, Lee TC, Chen WF, Yang KY (2013). Acute autologous bone flap infection after cranioplasty for postinjury decompressive craniectomy. Injury.

[15] Pathak A, Dhamande MM, Gujjelwar S, Das P, Chheda EV, Puthenkandathil R (2024). Fabrication of implant-supported auricular prosthesis using artificial intelligence. Cureus.

[16] Pathak A, Dhamande MM, Sathe S, Gujjelwar S, Khubchandani SR (2023). Revolutionizing maxillofacial rehabilitation for ocular defects: the impact of three-dimensional printing and sublimation transfer technique on changing horizons. Cureus.

[17] Pathak A, Dhamande MM, Sathe S, Gujjelwar S, Khubchandani SR, Minase DA (2023). Unveiling the realm of denture fabrication: revitalizing aesthetics and optimizing efficiency for geriatric patients. Cureus.

[18] Beri A, Pisulkar SK, Paikrao B, Bagde A, Bansod A, Shrivastava A, Jain R (2024). Quantitate evaluation of photogrammetry with CT scanning for orbital defect. Sci Rep.

[19] Robles LA, Morell A (2024). Autologous cranioplasty with bone flap preserved in conventional freezers: an adequate option in low resource settings. World Neurosurg.

[20] Shah AM, Jung H, Skirboll S (2014). Materials used in cranioplasty: a history and analysis. Neurosurg Focus.

[21] Woodhall B, Spurling RG (1945). Tantalum cranioplasty for war wounds of the skull. Ann Surg.

[22] Henry HM, Guerrero C, Moody RA (1976). Cerebrospinal fluid fistula from fractured acrylic cranioplasty plate. Case report. J Neurosurg.

[23] Blum KS, Schneider SJ, Rosenthal AD (1997). Methyl methacrylate cranioplasty in children: long-term results. Pediatr Neurosurg.

[24] Aydin S, Kucukyuruk B, Abuzayed B, Aydin S, Sanus GZ (2011). Cranioplasty: review of materials and techniques. J Neurosci Rural Pract.

